# Developing an appropriate depression and anxiety screening tool for use with Australian First Nations peoples living in the Torres Strait and Northern Peninsula Area of Australia: Protocol for a Delphi study

**DOI:** 10.1371/journal.pone.0292162

**Published:** 2023-12-07

**Authors:** Kathryn Meldrum, Valda Wallace, Torres Webb, Lynne Ridgway, Rachel Quigley, Edward Strivens, Sarah Russell

**Affiliations:** 1 College of Medicine and Dentistry, James Cook University, Cairns, Queensland, Australia; 2 North Coast Neuropsychology, East Ballina, New South Wales, Australia; 3 Cairns and Hinterland Hospital and Health Service, Cairns, Queensland, Australia; University of South Australia, AUSTRALIA

## Abstract

Tools that screen for depression and anxiety developed using the Western biomedical paradigm are still used with First Nations peoples globally, despite calls for cross-cultural adaption. Recent work by the research team found that tools used to screen for depression and anxiety were not appropriate for use with Australian First Nations peoples living in the Torres Strait and Northern Peninsula Area (NPA). of Australia. Consequently, the objective of this Delphi study is to gain consensus from an expert mental health panel to inform the development of an appropriate depression and anxiety screening tool(s). A Delphi study with Australian expert panellists will be used to reach consensus about whether an existing screening tool should be used or whether adaption or new tool development should take place. Three sequential rounds of anonymous online surveys will be used to reach consensus. The first round will seek consensus about the tool(s). Subsequent rounds will seek consensus on the development of the tool(s) identified in round one. Panellists will be identified using a combination of authorship of related publications, established national clinical or research profile in First Nations mental health, and/or by peer referral. Consensus will be reached when 75% of the panel agree. When agreement is not reached suggestions will be taken to the next round. If agreement is not achieved by the third round, the Steering Committee will make any outstanding decisions. Dissemination of the findings through continuing community engagement, conference presentations and publications will be led by Torres Strait Islander members of the research team.

## Introduction

Findings of a recent scoping review [1] identified that screening tools developed using the Western biomedical paradigm were used to screen for depression and anxiety in First Nations peoples globally. The use of these types of tools has been criticised because Indigenous worldviews and conceptualisation of health and wellbeing are different from the Western biomedical paradigm [2–5].

An understanding of First Nations people’s conceptualisation of health and well-being underpins the need to critique the appropriateness of using Western tools to screen for depression and anxiety [3, 6]. For example, Australia’s First Nations peoples holistic view of their health and well-being is interconnected and interrelated with their communities and Country [7]. Australian First Nations peoples use the term social and emotional wellbeing (SEWB), which is inclusive of Western conceptualisations of mental health [8]. Social and emotional wellbeing encompasses interactions between interrelated concepts that include the “body, mind and emotions, family and kinship, community, culture, country, and spirituality” [9 p.316]. As the concept of SEWB is multidimensional it is broader than the Western biomedical paradigm and focussed on a strengths-based approach to mental health [7]. In addition, language and terms used in screening tools developed using the Western biomedical model do not facilitate reporting of symptoms to ensure appropriate referral.

Since the mid-2000s Australian research teams have been active in cross-culturally adapting [10–12], validating [13, 14], and developing new tools to screen for depression [4, 15] and psychological distress (depression and anxiety) [16] for Australian First Nations peoples. The work conducted in partnership with Australian First Nations peoples acknowledges different expressions of depression and psychological distress as well as responding to the call for appropriate tools.

### Context for this study

The historical and continuing impacts of colonialism, including racism, affect the health and health-related outcomes of Australian First Nations peoples [17, 18]. People living in the Torres Strait (Zenadth Kes) and NPA may have their health impacted by the very remote location and concomitant effects that has on availability of health services, the high turnover of their personnel as well as the impacts of climate change [19]. Despite these impacts, 49% of First Nations Australians 15 years and over living in the region reported their health status as very good or excellent [20]. In contrast, 48% reported a current and long-term health condition and 19.6% over the age of 18 reported high psychological distress. However, the Australian Institute of Health and Welfare indicated that the psychological distress statistics should be viewed with caution due to up to 15% margin of error [20].

The context for this study is previous work undertaken by the research team, initially to determine the prevalence of dementia in the Torres Strait and NPA of Australia, between 2015–2018 [21]. During the dementia prevalence study, First Nations community members and health professionals identified that tools used to screen for depression and anxiety, the KICA-Dep [15] and Geriatric Anxiety Inventory [22] were not suitable in their current form [23].

The research team’s response to identifying that tools used to screen for depression and anxiety were inappropriate for First National peoples living in the Torres Strait and NPA was to seek funding for a project designed to develop screening tool(s) that are more appropriate. This broader project has been designed to be completed in four phases. These phases include:

1. Conduct yarning circles with community members and health professionals living in the Torres Strait and NPA.
2. Use a Delphi study to seek consensus on how to approach the development of a new screening tool(s).
3. Develop and pilot the new screening tool(s).
4. Validate the new screening tool(s).

Phase one, yarning circles, an Australian First Nations relational methodology and method [24–26], that relies on storytelling as a way of sharing knowledge, have already been completed. The yarning circles were focussed on sharing knowledge about words used to describe feelings of strong and low SEWB well the associated signs and symptoms. The protocol outlining how this phase was conducted was published in 2022 [27]. Phase one’s findings will be reported in a separate publication.

## Materials and methods

### Research aim and question

This protocol describes phase two, a Delphi study. The aim of this Delphi study is to seek the opinion of Australian mental health and/or SEWB experts about how to approach the third phase of the project, development and piloting of a depression and anxiety screening tool(s) for the Torres Strait and NPA regions of Australia. The research question that will guide the first round of the Delphi is: Given the findings of the yarning circles, should new tool(s) to screen for depression and anxiety be developed?

### Design

A Delphi technique (method or process) aims to use expert opinion to solve a research problem or to reach consensus about an identified issue [28, 29]. It is a well-used approach for developing guidelines [30], checklists [31] and policies [32] in the health sciences when studies using stronger evidence bases are not available or appropriate for the problem [28, 33]. A Delphi study systematically uses iterative surveys to seek the opinion of experts who participate anonymously [28, 30]. While the purpose of most Delphi studies is to seek consensus from an expert panel about the research problem, Häder [cited in 28 p. 3] identified three other types of Delphi’s: 1) Aggregation of ideas; 2) Most precise prediction of an uncertain issue; and 3) Collecting expert opinions on a diverse issue. Delphi studies have been used in mental health research to estimate prevalence, predict, “determine collective values” and “define foundational concepts” [33 p.889].

### Steering committee

A steering committee was convened to guide this Delphi study, some of whom are co-authors of this protocol as indicated by their initials in brackets. The Steering Committee is represented by members of an independent Knowledge Circle (VW, TW) (the research team’s Indigenous Reference Group), the ‘brain’s trust’ for the project who are non-Indigenous psychiatrists and psychologists (LR) with significant experience of working in the Torres Strait and NPA, as well as members of the research team, a neuropsychologist (SR) and the project lead (KM). VW and TW are also members of the research team. They are all located in Australia. The Steering Committee will meet remotely and initially discuss the progress of the project, the potential expert panel list, and protocol for the Delphi as well as contribute to and approve the first survey.

**Fig 1:**
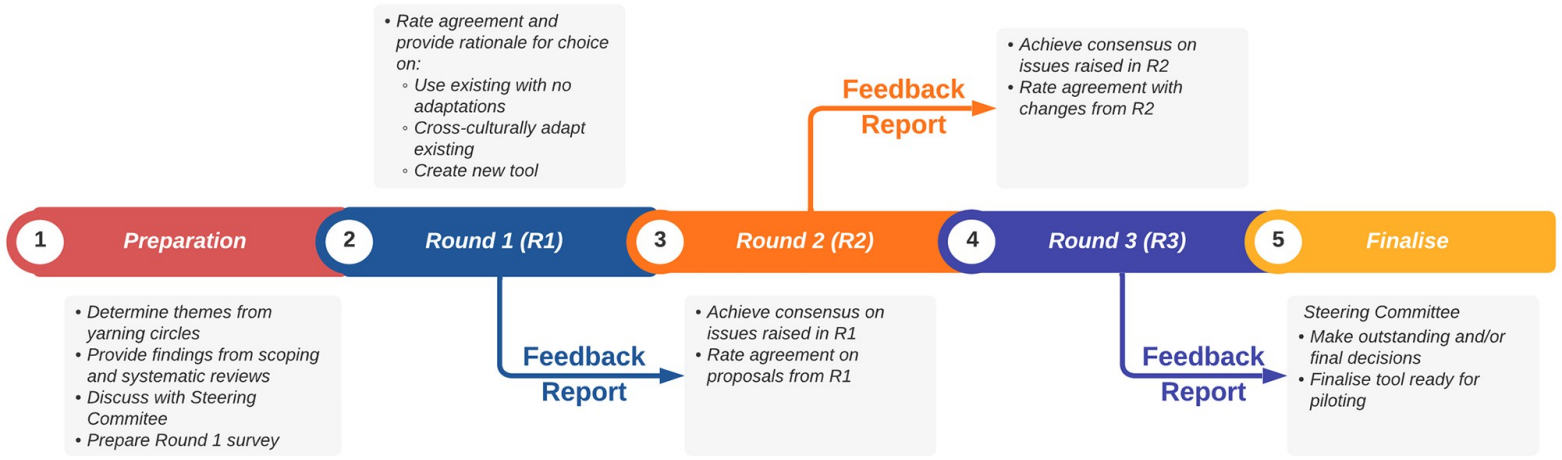
The overarching process for collecting data for this Delphi.

During the conduct of the Delphi study, the Steering Committee will review and analyse the responses to each survey. They will produce the associated feedback report prior to the next round in line with the process outlined in Fig 1 below. At the conclusion of the Delphi, the Steering Committee will make any outstanding decisions if consensus has not been reached on any issue and produce a final report. None of the Steering Committee members will act as panellists [31].

### Generation of questions for the Delphi rounds

Generation of questions used to develop the survey for the first Delphi round will be drawn from three sources. Firstly, findings from the yarning circles undertaken in the Torres Strait and NPA using the method described in the study protocol [27]. Relevant themes related to words used to describe signs and symptoms of depression and anxiety, as well as design suggestions for a tool, will be listed. Secondly, findings from a scoping review [1] that explored the range of tools to screen depression and anxiety in Indigenous peoples globally will be provided. Finally, the findings of a systematic review [34] that identified approaches to cross-cultural adaptation of standard tools, as well as the development of Indigenous tools, will be summarised. Findings of the scoping and systematic reviews will provide information about tools currently used in practice, as well as those cross-culturally adapted and/or developed for Australian First Nations peoples. Generation of questions for subsequent rounds will be guided by both consensus and/or issues raised in previous rounds, therefore cannot be identified in this protocol.

### Delphi panel selection (participants and recruitment)

Invitations will be extended to Australian clinicians, researchers and other experts including mental health and/or SEWB practitioners, mental health nurses, GPs, geriatricians, psychogeriatricians, psychiatrists, psychologists, and old age psychiatrists. Panellists will be identified using a combination of authorship of related publications, established national clinical and/or research profile in First Nations mental health/SEWB and/or by peer referral. Purposive and snowball sampling will be used to identify and recruit expert panellists. Potential panellists will initially be sent an introductory email inviting them to participate. The email will contain a short statement outlining the purpose of the project and ask them if they would like to participate. If they are unable to participate, they will be asked to suggest a colleague with similar expertise who could replace them. If a potential participant accepts their invitation, they will subsequently be sent an email with a link to the first Delphi round.

This Delphi aims to recruit a panel of up to 20 participants [35]. To achieve this number, an invitation will be extended to 40 clinicians and/or researchers, as it is acknowledged that not all will respond. The heterogeneity of the group will depend upon those who respond but invites will be emailed to a wide range of clinicians, researchers, and other experts from various locations across Australia that work in a variety of relevant roles. Panellists who complete at least one Delphi round will be asked if they wish to be acknowledged in the subsequent publication.

Ethics approval for this project was granted by the Far North Queensland Human Research Ethics Committee (HREC) (HREC/2021/QCH/73683-1518), James Cook University HREC (H8606) and Queensland University HREC (2022/HE000395).

### Inclusion/exclusion criteria

Panellists with knowledge of the topic area who have confirmed their willingness and suitability to participate will be included in the study.

### Data collection and analysis

The Qualtrics^™^ (Qualtrics, Provo, UT) platform will be used to design and disseminate the Delphi rounds. The overarching process for collecting data for this Delphi is outlined in Fig 1.

This three-round Delphi study will be conducted across three months. Each Delphi round will be open online for up to two weeks. If necessary, a reminder email will be sent approximately seven days before the associated Delphi round is closed. A further two weeks will be used to analyse the data, provide a feedback report to the Steering Committee and prepare the subsequent round’s survey.

The research question for the Delphi will guide the first round. Consequently, the expert panel will be asked: Given the findings of the yarning circles, should new tool(s) to screen for depression and anxiety be developed? This round will seek consensus on whether:

1. Existing screening tools can be used.
2. Cross-cultural adaptation of existing tool(s) should be undertaken; or
3. A new tool(s) should be developed.

Experts who agree that existing screening tools should be used will be asked to recommend a tool(s). Conversely, experts who do not agree will be asked whether cross-cultural adaptation or a new tool should be developed. If this choice is made by experts, then they will be asked about their preference for integrating the findings of the previous (yarning circles) phase of the broader study. Response options will range from 5-item Likert scale ranging from 1 (strongly disagree) to 5 (strongly agree) with 3 being ‘no opinion’. The response option ‘outside my expertise’ will also be provided for each statement/question [31]. Where a 5 item Likert scale is used across any of the rounds the same response options will be provided. In addition, for some statements, experts will be presented with a list of choices to select from. The expert panel will also be provided with opportunities to provide a rationale for their choice(s) in free text boxes after each question and across all rounds.

At the commencement of round two, experts will be provided with a report containing results of the previous round. Overall results for each item will be presented with analysis including percentages, frequency distributions, measures of central tendency (mean, median, mode) and levels of dispersion (standard deviation and interquartile range) where relevant. ‘Outside my expertise’ responses will not be included in the data analysis or in the consensus calculation for any of the rounds. Quantitative data will be analysed using SPSS^™^ (IBM International) Statistical program. Qualitative responses will be analysed using thematic analysis [36] using NVivo^™^ (QSR International) to manage the data.

The initial focus of round two will be to move towards achieving consensus on any issues raised in round one. Experts will be asked to rate their agreement with the issues. If there are no outstanding issues, experts will be asked to rate their agreement with the outcomes of round one. Proposals for round two may include delivery approach if there was consensus that cross-cultural adaption or a new tool should be developed. A 5-item Likert scale as well as a free text box at the end of each question will ask participants for further comments about the reasons why they agree or disagree with the findings and rationale for their decision as well as any comments on further additions/omissions that they would like to suggest.

A report detailing the outcomes of round two will again be presented to the experts at the commencement of round three. Data from round two will be analysed in the same way as round one. As with round two, initially consensus will be sought on the findings from the previous round. Response options for the associated statements/questions will be a 5-item Likert scale.

At the conclusion of data collection for round three a final report will be prepared for the Steering Committee. If consensus was not reached in round three of the Delphi the Steering Committee will make the necessary decisions.

### Ethical considerations

The information sheet and consent process will precede the first Delphi round survey. Once consent has been given by the participant, they will be able to access the round one survey. Panellist responses will be anonymous, and all participants will be blinded to the responses of others. General feedback on the responses from rounds one and two will be provided. All data will be downloaded and kept on a password protected computer in a locked office. Only specified members of the Steering Committee (KM, SR) will have access to the raw data. Data will be deleted after five years in accordance with standard ethical guidelines.

#### Personal and public involvement and engagement

This Delphi study is phase two of a four-phase study aimed at developing an appropriate tool(s) for screening depression and anxiety in Australian First Nations peoples living in the Torres Strait and NPA. The need for a new tool(s) arose from community and health professional feedback during and after the dementia prevalence study [21, 23]. The research team also works with an existing independent Knowledge Circle (Indigenous Reference Group) that oversees research in the region. The Knowledge Circle comprises Aboriginal and Torres Strait Islander community members, aged care workers, healthcare staff and academics who are committed to supporting the health of people in their communities. Projects are also conducted in partnership with local health and aged care providers in the Torres Strait and NPA. This Delphi study is guided by the Knowledge Circle and the Steering Committee which also contains Knowledge Circle members.

## Discussion

This study will support the development of an appropriate tool(s) to screen for depression and anxiety in Australian First Nations peoples living in the Torres Strait and NPA. It responds to previous research that identified that existing tools were not appropriate for this population [23]. While a Delphi study is seldom used to develop screening tools [28], it has been used in mental health research [33] and to develop questions for a geriatric depression inventory appropriate for the Chinese culture [37].

A Delphi process is appropriate for this study because it draws on the opinion of clinicians, researchers and other experts working in the field. The approach of this Delphi study is a combination of two different types according to the definition of Häder [cited in 28, p. 3]: Type 1—The aggregation of ideas; and Type 4—Consensus. Features of the type one approach include qualitative data collection, generating ideas that answer the research problem and selecting a small number of interdisciplinary experts. In contrast, type 4 uses quantitative data collection to achieve the highest rate of consensus with a range of experts that may include interest groups and members of the public.

The design of this Delphi study seeks to gather and summarise different ideas about how to solve the research problem [28]. Type 4 also applies to the design of this Delphi because a high percentage of agreement has been determined (75%) *a priori*. Also, invitations to participate in the Delphi study will be based on expertise and draw on a range of groups, not just clinicians working in the mental health/SEWB field. Mixed methods will also be used to gather information across the Delphi rounds which combines type 1 (qualitative) and type 4 (quantitative) data collection and analysis approaches.

### Policy and practice implications

The outcome of the broader four-phase project will produce a more appropriate depression and anxiety screening tool for First Nations peoples living in the Torres Strait and NPA. It is hoped that the new screening tool will be adopted by primary health and geriatric settings in the region. Although this cannot be guaranteed. In addition, the outcomes of the broader project align with a federally funded project focussed on developing a healthy ageing framework for the Torres Strait. The Healthy Ageing Framework for the Torres Strait project aims to provide a suite of resources to support primary health centres in the region. It is anticipated that the new screening tool will be part of this suite of resources.

### Limitations

There are two identified limitations for this Delphi study. Firstly, the opinion of panellists who agree to participate may differ from those that are unable to participate. To limit the potential impact of this issue, it is hoped that an initial invitational email will enable the research team to establish who is able to participate and subsequently determine whether additional invitations need to be made. In addition, panellists who are unable to participate will be asked to suggest other participants with similar backgrounds that may be able to replace them on the panel. Secondly, panellist drop-out from round-to-round is a potential limitation, especially when the rounds are conducted over a long period of time and are expecting a large time commitment [31]. To decrease the impact of this limitation, Delphi rounds will only be open for two weeks with a two week turn-around between each. Moreover, each Delphi survey will be relatively short with ‘pathways’ set-up for each panellist depending on their response to statements/questions which will decrease the total time spent answering questions.

### Dissemination plan

Results of this study will be led by Torres Strait Islander members of the research team. Results will be disseminated using a range of strategies including ongoing community engagement, conference presentations and publications. For example, the research team regularly spends time in Torres Strait Island communities and consequently conducts ‘rolling’ community engagement where the progress of projects currently underway or planned are shared and discussed. In addition, project progress is regularly shared with Torres Strait peak bodies such as Torres Strait Regional Authority, Torres Strait Island Regional Council, Gur A Baradharaw Kod (GBK) Torres Strait Sea and Land Council, and Torres Shire Council through deputations at meetings and informal conversation. Finally, conference presentations and publications will be guided by relevant guidelines such as the Guidance on Conducting and Reporting Delphi Studies (CREDES) [30] and/or any other relevant guidelines [28, 38].

## Supporting information

S1 File Responses to Plos One’s inclusivity on global research questionnaire. (PDF)
