# Supplementary material for: Developing an appropriate depression and anxiety screening tool for use with Australian First Nations peoples living in the Torres Strait and Northern Peninsula Area of Australia: Protocol for a Delphi study
Source: PLoS One. 2023 Dec 7;18(12):e0292162. doi: 10.1371/journal.pone.0292162 (PMC10703283; doi:10.1371/journal.pone.0292162)
Supplement: S1 File — (PDF) [file pone.0292162.s001.pdf]

Inclusivity in global research

PLOS' policy on inclusivity in global research aims to improve transparency in the reporting of research performed outside of researchers' own country or community and ensures that PLOS publications reporting global research adhere to high standards for research ethics and authorship. Authors of relevant research articles may be asked to complete the questionnaire below, which outlines ethical, cultural, and scientific considerations specific to inclusivity in global research. This questionnaire may be requested when researchers have travelled to a different country to conduct research, if research uses samples collected in another country, research with Indigenous populations or their lands, or if research is on cultural artefacts. Researchers travelling to another country solely to use laboratory equipment will not normally be required to complete the questionnaire. However, the questionnaire can be requested at the journal's discretion for any submission – if you have been requested to complete this questionnaire by the PLOS journal you submitted to, please do so.

Please complete the questionnaire below and include this as a Supporting Information file with your manuscript. Note that if your paper is accepted for publication, this checklist will be published with your article in the supporting information files. Please ensure that you reference the checklist in the main body of your manuscript. We suggest adding a subsection 'Inclusivity in global research' to your Methods section and adding the following sentence: "Additional information regarding the ethical, cultural, and scientific considerations specific to inclusivity in global research is included in the Supporting Information (SX Checklist)"

The questions have been designed to be applicable to a wide range of study types, and there are subsections for both human subjects research and non-human subjects research. If any of the questions are not relevant to your research please mark them as "N/A" as appropriate.

Ethical considerations, permits and authorship

This section is applicable to all research types.

Provide details as to who granted permissions and/or consent for the study to take place in the Methods section of your manuscript. This should include the names of **all** ethics boards, governmental organizations, community leaders or other bodies that provided approval for the study. If individuals provided approval refer to these people by their role or title but do not list their name(s).

Reported on page number: 8

If there were any deviations from the study protocol after approval was obtained please provide details of these changes in the Methods section of your manuscript.

Reported on page number: Not applicable

Did this study involve local collaborators that are residents of the country where the research was conducted or members of the community studied? If you do not have any authors from said communities, please provide an explanation for this below.

Yes

Everyone listed as an author should meet PLOS' criteria for authorship and all individuals who meet these criteria should be included in the author byline, rather than the acknowledgements. Authorship criteria is based on the International Committee of Medical Journal Editors (ICMJE) Uniform Requirements for Manuscripts Submitted to Biomedical Journals - for further information please see here: https://journals.plos.org/plosone/s/authorship.

Human subjects research (e.g. health research, medical research, cross-cultural psychology)

Did you obtain written informed consent from a representative of the local community or region before the research took place? How did you establish who speaks for the community? Details of written informed consent obtained from study participants should be reported separately in the Methods section of your manuscript.

This manuscript reports the protocol for a study that does not directly involve grass roots community members.

The information sheet and consent process will precede the first Delphi round survey. Once consent has been given by the participant, they will be able to access the round one survey. Panellist responses will be anonymous, and all participants will be blinded to the responses of others. General feedback on the responses from rounds one and two will be provided. All data will be downloaded and kept on a password protected computer in a locked office. Only specified members of the Steering Committee (KM, SR) will have access to the raw data. Data will be deleted after five years in accordance with standard ethical guidelines.

How did members of the local community provide input on the aims of the research investigation, its methodology, and its anticipated outcome(s)?

This Delphi study is phase two of a four-phase study aimed at developing an appropriate tool(s) for screening depression and anxiety in Australian First Nations peoples living in the Torres Strait and NPA. The need for a new tool(s) arose from community and health professional feedback during and after the dementia prevalence study. The research team also works with an existing independent Knowledge Circle (Indigenous Reference Group) that oversees research in the region. The Knowledge Circle comprises Aboriginal and Torres Strait Islander community members, aged care workers, healthcare staff and academics who are committed to supporting the health of people in their communities. Projects are also conducted in partnership with local health and aged care providers in the Torres Strait and NPA. This Delphi study is guided by the Knowledge Circle and the Steering Committee which also contains Knowledge Circle members.

When engaging with the local community, how did you ensure that the informed consent documents and other materials could be understood by local stakeholders?

Not applicable to this study.

Will the findings of the research be made available in an understandable format to stakeholders in the community where the study was conducted (e.g. via a presentation, summary report, copies of publications, etc.)? Please provide details of how this will be achieved.

Ongoing community engagement through informal discussions, formal prsentations, presentations at meetings of peak bodies in the region such as Torres Strait Regional Authority, Torres Strait Island Regional Council, Gur A Baradharaw Kod (GBK) Torres Strait Sea and Land Council, and Torres Shire Council.

Non-human subjects research using specimens/ animals collected as part of the study, or those housed in archival collections. Examples include archaeology, paleontology, botany and zoology.

Did the permission you obtained from a local authority to perform the study include an agreement on access to outputs and benefit sharing? This may include procedures to enable fair distribution of the benefits and resources arising from the research performed. Please include any details of Prior Informed Consent and

Not applicable to this study.

Benefit Sharing Agreements obtained. These may be required by field-specific regulations, for example the Convention on Biological Diversity (CBD) and the associated Nagoya Protocol.

If the material used in your study was imported, please A) provide the year it was imported and B) indicate whether permits were obtained to import/export the materials used, C) provide details of any permits obtained. If this information is not available, please indicate this.

Not applicable to this study.

If you used archival specimens, please state how the material used in your study was acquired by the institute it is held in and provide details of any permits obtained for the original excavations/ sample collection. If this information is not available, please indicate this.

Not applicable to this study.

How was the potential cultural significance of the materials collected in your study to local communities considered in your research design? Were Indigenous peoples and/or local researchers and institutions involved with archaeological excavations / collection of specimens? If so, please provide a description of their involvement.

Not applicable to this study.

If your manuscript includes photographs of human remains please indicate whether authors obtained permission from descendants or affiliated cultural communities to do so.

Not applicable to this study.
